# Development and Effects of Mobile-Application-Based Parenting Support Program for Premature Infants’ Mothers

**DOI:** 10.3390/healthcare11192639

**Published:** 2023-09-27

**Authors:** Hye Young Ahn, Hyun Jeong Ko, Hee Jee Jo

**Affiliations:** College of Nursing, Eulji University, Uijeongbu 11759, Republic of Korea; 20080039@eulji.ac.kr

**Keywords:** premature infants’ mothers, app-based program, parenting confidence, parenting support, premature infants

## Abstract

Background: Premature infants are separated from their parents after birth and are admitted to the neonatal intensive care unit. Separation of babies from mothers causes various problems. Therefore, as the number of premature infants increases every year, educational programs to continuously support baby care are needed. Methods: In this study, a nonequivalent control group non-synchronized design was employed. The subjects of the study were 52 mothers of premature infants (16 in the app-based program group, 18 in the electronic document group, and 18 in the control group) using a portal site for parents from February to April 2023. The app-based program and electronic document group followed a parenting support program for two weeks. Results: There were significant differences in maternal confidence between the app-based, electronic document, and control groups (F = 7.354, *p* = 0.002). Conclusions: Providing necessary information and emotional support through professional counseling centers’ app-based programs for premature infants’ mothers, and providing interaction through member community bulletin boards, are proposed to form an effective educational program.

## 1. Introduction

In 2021, a total of 23,800 premature infants born in South Korea at less than 37 weeks of gestation represented 9.2% of all the births, 0.7% up from the previous year [1]. In 2022, the World Health Organization noted that approximately 15 million infants are now born prematurely each year, which means that at least 1 out of 10 births is premature [2]. For this reason, premature infants’ parents, medical staff, and relevant experts and institutions highlight that it should be a priority to improve the existing education and support for premature infants’ parents [3].

Most premature infants are institutionalized in neonatal intensive care units (NICUs) to receive special treatment from medical staff right after birth according to their gestational age, weight, and condition [4]. It has been reported that many parents suffer from stress during their infant’s stay in the NICU [5,6,7]. In particular, mothers can suffer from mental distress after giving birth to a premature infant [8] and experience negative emotions such as stress, anxiety, anger, and depression due to their infant’s gestational age, weight, long-term hospitalization right after birth, and so on [9].

Separation of parents from infants who are hospitalized can adversely affect both of them [10]. Unable to lead in decision-making on their newborn’s treatment, they can feel they have lost control and have difficulty in forming a parental identity [11,12]. Such a situation can prevent them from performing maternal roles effectively; therefore, intervention aimed at positive changes in maternal identity is required [13]. In addition, the COVID-19 pandemic caused most of the NICUs to place restrictions on visits in order to protect the infants from the risk of infection all around the world [14,15]. This situation increased parents’ anxiety and mental distress; furthermore, long-term separation from their newborn not only made it difficult for many parents to become attached but also hampered the infant’s growth and development [16].

Premature infants are developmentally behind and are at a relatively higher risk of being exposed to potential harm, compared with term infants, in terms of growth and development due to diverse complications, including some possibly caused by the course of treatment [17].

Most premature infants’ parents attach great importance to any information that the NICU medical staff provides regarding their infant. However, they may not always receive desirable information. Often, premature infants’ parents attempt to obtain additional information through other types of medical services, written teaching materials, or Internet searches [18]. More recently, they have begun to use social media, search engines, and mobile applications to obtain information about their newborn’s health; in particular, young parents tend to prefer using mobile applications [19,20,21]. Due to the worldwide COVID-19 pandemic having normalized noncontact education, the development of a smartphone-based education application may be an effective way of providing information to premature infants’ parents [22].

In the USA and Europe, educational mobile applications are already being actively developed for parents of premature infants. Research involving a systematic literature review of mobile app assessment in parents of premature infants staying in NICUs examined 27 applications retrieved from the Google and Apple app stores [23]. Other international and domestic studies include a study confirming the effects of a constant nutrition control intervention based on a mobile healthcare application on the growth and development of premature infants [24], a study on the development of a smartphone education app for premature infants’ parents [25,26], and a study on an intervention program for premature infants’ parents [27,28,29]. South Korea currently lacks app-based education programs for premature infants’ mothers when compared with other countries. Most of the existing intervention programs provide a one-session program, not long-term education provided over all stages of care. Furthermore, the majority of existing app-based education programs focus either on premature infants’ health problems or on their growth and development, while very few of them involve emotional support for their mothers.

Considering that the birthrate of premature infants increases on an annual basis, the issue of their mothers’ emotional distress is timely to resolve. Accordingly, this study aimed to develop and implement a parenting support app-based education program that permits premature infants’ mothers to participate actively in care from the early stage of childrearing, provides them with emotional support, and provides professional information about infants, and assess its usefulness.

### Hypotheses

The research hypotheses on the effects of a parenting support app-based education program for premature infants’ mothers were as follows:

(1) There will be differences in the maternal identity of mothers between the app-based program group, electronic document group, and control group.
(2) There will be differences in maternal postpartum attachment between mothers in the app-based program group, electronic document group, and control group.
(3) There will be differences in maternal confidence between mothers in the app-based program group, electronic document group, and control group.

## 2. Materials and Methods

### 2.1. Research Design

This study used a nonequivalent control group non-synchronized design to develop and apply a parenting support app-based education program for premature infants’ mothers and confirm differences in its effectiveness through comparison with a parenting support PDF-based education group and a control group.

### 2.2. Subjects

**Figure 1:**
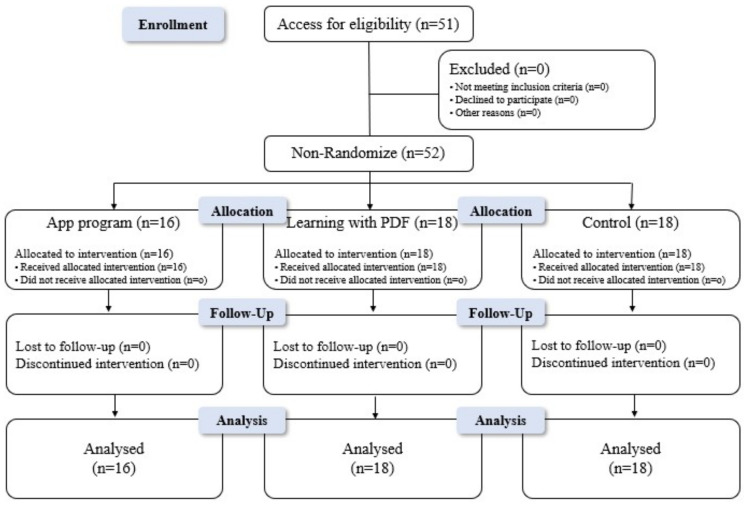
Process flow diagram.

The sample size was estimated using a G*Power 3.1.9.7 program. The sample size for the F-test was 51 persons, with the significance level of α = 0.05, testability (1–β) of 0.8, and effect size of 0.45 [30]. A total of 52 respondents were finally chosen: 16 in the app-based education program group, 18 in the electronic document group, and 18 in the control group. To prevent the experiment being compromised by communication between groups, data analysis was not synchronized between the control group and the experimental groups, as shown in Figure 1.

The subjects were recruited by posting a notice advertising for participants on the bulletin boards of two portal sites for parents (Mom’s Holic Baby and Beautiful Preterm Babies).

The inclusion criteria for subjects were as follows:

- Mothers of premature infants who were born less than a month before at 31–37 weeks of gestational age and who weighed 1.5–2.5 kg at birth;
- Mothers of premature infants who were staying in the NICU or being given care at home;
- Mothers capable of using a smartphone and with Wi-Fi or the necessary data;
- Mothers who understood the purpose of this study and agreed to participate.
- The exclusion criteria for subjects are as follows:
- Premature infants with congenital malformations or severe complications;
- Mothers requiring treatment for postpartum complications and having their daily life restricted.

### 2.3. Instruments

#### 2.3.1. Maternal Identity

A semantic differential scale, developed by Osgood and colleagues [31] and improved by Walker [32], was used to measure the accessible area of mothers’ maternal identity. This instrument was translated and revised by Kim and Tak [33] to apply in this study. It is a 7-point scale with 11 items, scoring between 11 and 77, with a higher score meaning more positive maternal identity. For reliability, Cronbach’s α was estimated at 0.89 in Kim and Tak [33]. For reliability of internal consistency in this study, Cronbach’s α was 0.92.

#### 2.3.2. MPAS: Maternal Postpartum Attachment Scale

Kim and Tak produced the Korean version of the maternal postpartum attachment scale (MPAS-K) [34], originally developed by Condon and Corkindale (MPAS) [35], by testing it for validity and reliability in mothers of infants and toddlers less than 36 months of age who had stayed in NICUs. It is a Likert scale scoring 2–5 for each of 14 items in total. To give identical weight to each item, recoding is recommended with 1;5 for a 2-point scale, 1;3;5 for a 3-point scale, and 1;2.3;3.6;5 for a 4-point scale. The higher the score, the more positive the maternal attachment. For reliability, Cronbach’s α was 0.80 for all items in Kim and Tak [33]. For reliability of internal consistency in this study, Cronbach’s α was 0.81.

#### 2.3.3. Maternal Confidence

The maternal confidence questionnaire was developed by Parker and Zahr [36] to measure parenting confidence, which is the ability of mothers to use their parenting skills to perceive an infant’s needs. It was then translated by Choi and Shin [37]. This instrument is composed of a total of 14 items: 12 positive ones and 2 negative ones. It is a 5-point scale, scoring from 1 totally disagree to 5 totally agree, with total score of 14–70; the higher the score, the more positive the parenting confidence. Cronbach’s α was estimated at 0.92 in Choi and Shin [37]. For reliability of internal consistency in this study, Cronbach’s α was 0.87.

#### 2.3.4. Educational Satisfaction

The experimental group was given interventional education and had their satisfaction measured using the instrument developed by the researcher. This instrument is composed of a total of 7 items concerning the understanding of the app-based education program and electronic documents, motivation, clarity of data, technical stability of the given program, and achievement after completing an intervention. It is a 5-point scale, with a higher score meaning more positive educational satisfaction. For reliability of internal consistency in this study, Cronbach’s α was 0.60.

### 2.4. Data Collection

This study obtained approval from the Institutional Review Board of Eulji University (EU22-87) in Uijeongbu, Gyeonggi Province. The data were collected from 1 February to 30 April 2023. A notice was posted advertising for this study on portal sites for parents (Mom’s Holic Baby and Beautiful Preterm Babies), and a total of 52 persons were recruited: 16 in experimental group I (app-based education program group), 18 in experimental group II (electronic document group), and 18 in the control group. The subjects who were willing to participate in the study and who met the criteria were asked to read the written consent and decide on agreement.

### 2.5. Development of Parenting Support Education Program for Premature Infants’ Mothers

The intervention method in this study, or the parenting support app-based education program for premature infants’ mothers, was developed by using the ADDIE model, that is, through the stages of analysis, design, development, implementation, and evaluation, along with an app professional’s advice.

#### 2.5.1. App-Based Education Program

At the analysis stage, a literature review was performed to analyze the existing education programs, websites, and applications for premature infants’ parents. Keywords were used to search the Apple App Store and Google Play from 27 January 27 to 25 February 2023. A total of 9 keywords—premature infant, preterm infant, premature infant’s parent, premature infant’s mother, neonatal ICU, neonatal intensive care unit, premature infant caring, prematurely born baby, and NICU—brought up 4 app results, with the exception of those irrelevant to this study. Since South Korea has only a few apps for premature infants’ mothers, the keyword “newborn” was added to extend the search. Finally, 20 apps associated with the theme of the study were selected. Of these apps, 7 apps (35%) provided information about growth and development, 10 (50%) were related to breastfeeding and feeding, 7 (35%) provided health information, 2 (10%) operated membership communities, 1 (5%) provided information about physical development, and 8 (40%) provided a growth curve.

At the design stage, the structure of the app program was determined. There were five large categories within the theme: the first category is related to baby growth, with such details as life in hospital, breastfeeding, formula-feeding, defecation, kangaroo care, coping tips for an emergency, and programs for supporting premature infants; the second category is related to baby development, with such details as development milestones, intervention information, and growth curve graphs; the third category is related to record-keeping concerning breastfeeding, formula-feeding, defecation, a parenting diary, and so on; the fourth category is related to emotional support given to mothers, which involves a ‘professional counseling center’ aimed at two-way communication for the researcher to give technical knowledge and information when asked a question about baby care, in addition to a membership community; the fifth category is to give a log-in function and personalized communications.

**Figure 2:**
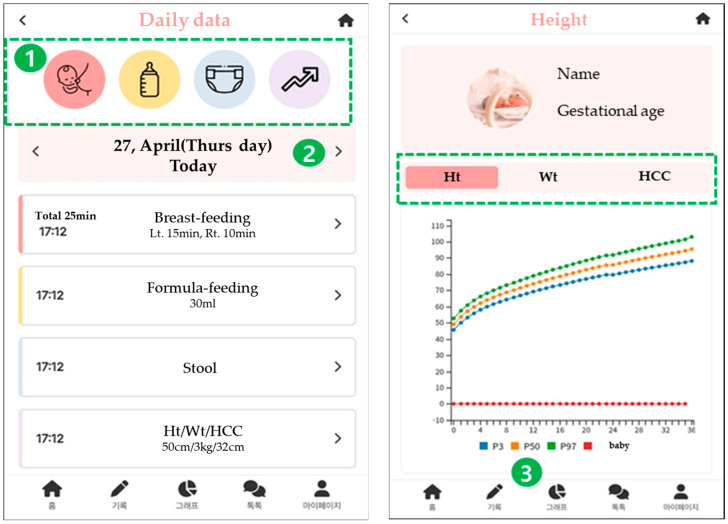
App screen development diagram.

At the development stage, the operating system was developed in a hybrid form so that it could be usable both in Android and in iOS. The screen for the parenting support app-based program for premature infants’ mothers is as shown in Figure 2.

After its development, the parenting support app for premature infants’ mothers was tested for content validity by experts. It was tested by a total of 6 experts: 2 professors in pediatric nursing, 3 nurses with at least ten-year careers working in NICUs, and 1 professor in pediatrics. As a result of content validation of 17 items, the CVI index was estimated at the average level of 0.94, which met the reference level of 0.80 or higher [38].

At the implementation stage, participants received an explanation about how to use the app and received membership with their ID and password. They were asked to use the app at least three times a week, and the researcher checked their log-in status through the administrator’s page from time to time.

At the assessment stage, the experts and the subjects were asked to give opinions in terms of app composition assessment, and the app was finally developed following its revision and supplementation.

#### 2.5.2. PDF Teaching Materials

The teaching materials in the form of electronic documents for the other experimental parenting support education program for premature infants’ mothers have identical contents to the app program, which was converted to the electronic document (PDF) format by using Adobe Acrobat Reader. Parts of the app program such as the operable professional counseling centers, communities, parenting diary, and recording (amount of feeding, defecation, growth curve graph) were excluded.

#### 2.5.3. Experimental Treatment

To prevent the experiment being compromised by communication between groups, data were first collected from the control group. The pretest involving a survey of the general characteristics of premature infants and their mothers, whereby maternal identity, maternal postpartum attachment, and parenting confidence were investigated in the control group. A posttest was performed two weeks after the pretest. After data collection from the control group, a pretest was performed in experimental group 2 (electronic document group). Immediately after the pretest, experimental group 2 (electronic document group) was given the PDF teaching materials and, two weeks later, underwent the posttest.

**Figure 3:**
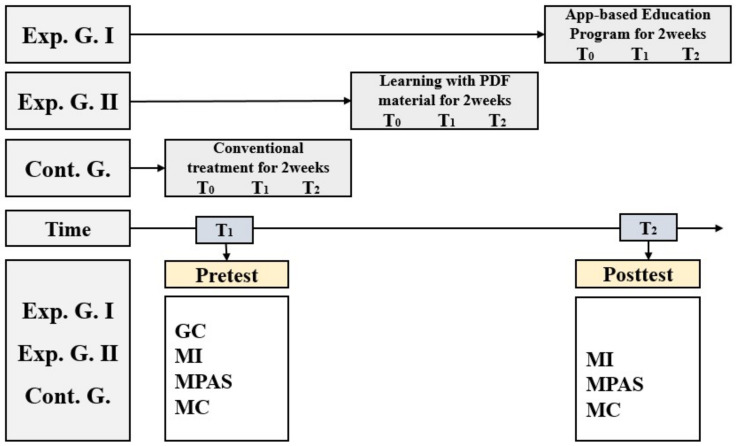
Data collection procedure flow diagram.

After data collection from experimental group 2 (electronic document group), a pretest was performed in experimental group 1 (app-based education program group). Immediately after the pretest, the app-based education program was provided and, two weeks later, a posttest was performed. Overall, the data collection procedure was as follows *(*Figure 3).

### 2.6. Data Analysis

The collected data were analyzed using the IBM SPSS Statistics 27.0 program. The respondents’ general characteristics were presented in frequency, percentage, and the mean and standard deviation. The chi-square test, *t*-test, and one-way ANOVA were used to test the general characteristics for homogeneity among the three groups. One-way ANOVA was performed for homogeneity in the dependent variables among the three groups. One-way ANOVA was then used to compare the dependent variables among the mothers in the three groups in an attempt to confirm the effectiveness of the app-based education program. Those variables the pretest found to be inhomogeneous were treated as covariates and analyzed with ANCOVA. The Scheffe test were used in the posttest. A *t*-test was applied to analyze the differences in educational satisfaction between the two experimental groups.

### 2.7. Ethical Considerations

This study was approved by the Institutional Review Board of Eulji University (EU22-87) in Uijeongbu, Gyeonggi Province. The subjects were given an explanation about the purpose and contents of the study and their confidentiality. We ensured their willingness to participate, and the study was conducted with voluntary consent. They were informed that they could withdraw at their will any time, that their participation would be kept confidential, and that their responses would never be used for anything other than the purposes of this study. They were also told that their personal information collected for the study would be regarded as confidential, and the collected data would be discarded three years after being kept with an identification code. After the end of the experiment, the control group and the electronic document group were also given information about the app-based education program, to use if they wanted.

## 3. Results

### 3.1. Pretest for Homogeneity in Participants

#### 3.1.1. Homogeneity Test for General Characteristics of Premature Infants’ Mothers

A total of 52 respondents were chosen: 16 in the app-based education program group, 18 in the electronic document group, and 18 in the control group. For the general characteristics, the results were as show in Table 1. In terms of their homogeneity among the three groups, no significant inter-group difference was found in age, education level, monthly income or childbirth experience.

#### 3.1.2. Homogeneity Test for General Characteristics of Premature Infants

The results of the homogeneity test for the general characteristics of premature infants were as shown in Table 2. The gestational age, duration of stay in hospital, gender, delivery type, experience of oxygen therapy, experience of treatment with a ventilator, birth height, weight, and head circumference were homogeneous, with no significant difference among the three groups.

#### 3.1.3. Pretest for Homogeneity in Dependent Variables of Premature Infants’ Mothers

For maternal identity, the pretest found that premature infants’ mothers scored 58.88 ± 5.18 in the app-based education program group, 51.39 ± 13.40 in the electronic document group, and 61.67 ± 7.14 in the control group. For maternal postpartum attachment, the pretest found that they scored 59.84 ± 4.09 in the app-based education program group, 56.39 ± 9.36 in the electronic document group, and 61.48 ± 3.41 in the control group. For parenting confidence, the pretest found that they scored 53.81 ± 8.78 in the app-based education program group, 50.33 ± 8.15 in the electronic document group, and 53.06 ± 6.49 in the control group.

As for homogeneity in the dependent variables of the premature infants’ mothers, the pretest found no statistically significant difference in maternal postpartum attachment or parenting confidence, though there was one in maternal identity (F = 5.735, *p* = 0.006) among the three groups (Table 3).

### 3.2. Hypothesis Testing

#### 3.2.1. Hypothesis 1

“Maternal identity will differ among the app education program, electronic document, and control groups”.

The pretest found significant differences in maternal identity: 58.88 ± 5.18 in the app-based education program group, 51.39 ± 13.40 in the electronic document group, and 61.67 ± 7.14 in the control group (F = 5.735, *p* = 0.006). With maternal identity as a covariate in the pretest, the posttest found no significant difference in maternal identity: 62.56 ± 6.00 in the app-based education program group, 55.61 ± 14.56 in the electronic document group, and 61.00 ± 6.33 in the control group (F = 1.550, *p* = 0.223).

There was no significant inter-group variation in maternal identity: 3.69 ± 4.09 in the app-based education program group, 4.22 ± 8.13 in the electronic document group, and −0.67 ± 6.84 in the control group (F = 2.884, *p* = 0.065). Therefore, Hypothesis 1 “maternal identity will differ among the app education program, electronic document, and control groups” was rejected.

**Figure 4:**
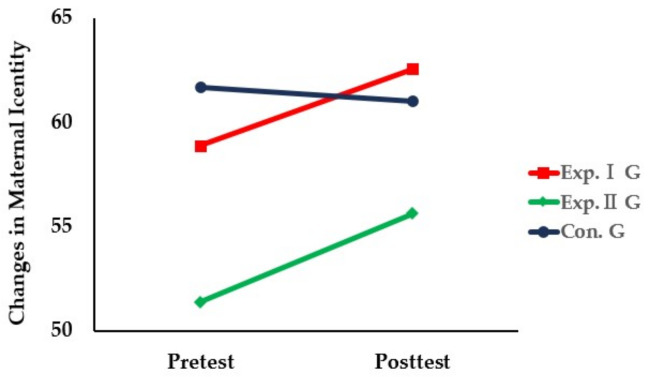
Comparison of changes in maternal identity among three groups.

The results of the comparison of maternal identity among the three groups before and after the intervention using the app-based parenting support education program for premature infants’ mothers were as follows (Table 4, Figure 4).

#### 3.2.2. Hypothesis 2

“Maternal postpartum attachment will differ among the app education program, electronic document, and control groups”.

The pretest found no significant difference in maternal postpartum attachment: 59.84 ± 4.09 in the app-based education program group, 56.39 ± 9.36 in the electronic document group, and 61.48 ± 3.41 in the control group (F = 3.056, *p* = 0.056). The posttest found no significant difference in maternal postpartum attachment: 63.93 ± 3.57 in the app-based education program group, 60.28 ± 6.89 in the electronic document group, and 61.69 ± 4.58 in the control group (F = 2.064, *p* = 0.138).

The posttest found that maternal postpartum attachment differed significantly among the three groups: 4.09 ± 3.72 in the app-based education program group, 3.88 ± 5.02 in the electronic document group, and 0.22 ± 3.69 in the control group (F = 4.716, *p* = 0.013). The posttest found that the app education program and electronic document groups scored higher for the differences in postpartum maternal attachment than the control group. Therefore, Hypothesis 2 “postpartum maternal attachment will differ among the app-based program, electronic document, and control groups” was partially supported.

**Figure 5:**
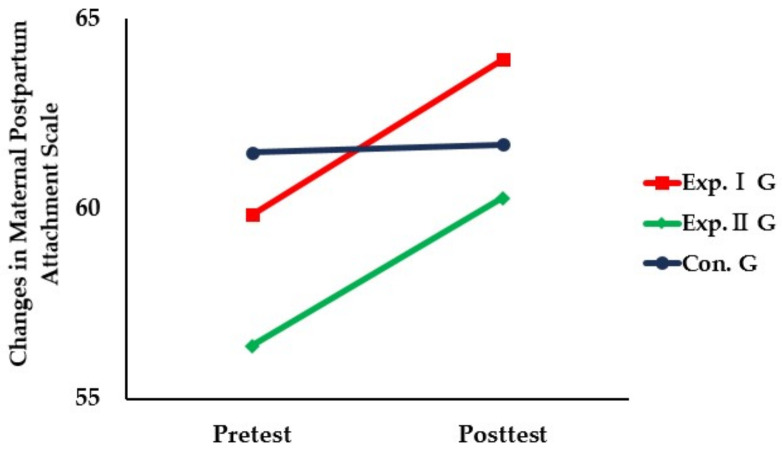
Comparison of changes in maternal postpartum attachment scale among three group.

The results of the comparison of maternal postpartum attachment among the three groups before and after the intervention using the app-based parenting support education program for premature infants’ mothers were as follows (Table 5, Figure 5).

#### 3.2.3. Hypothesis 3

“Maternal confidence will differ among the app education program, electronic document, and control groups”.

The pretest found no significant difference in parenting confidence: 53.81 ± 8.78 in the app-based education program group, 50.33 ± 8.15 in the electronic document group, and 53.06 ± 6.49 in the control group (F = 0.949, *p* = 0.394). The pretest found significant differences in parenting confidence: 58.56 ± 5.72 in the app-based education program group, 51.72 ± 6.53 in the electronic document group, and 51.94 ± 5.19 in the control group (F = 7.354, *p* = 0.002). The posttest found that the app education program scored higher for parenting confidence than the electronic document and control groups.

The posttest found that parenting confidence differed significantly among the three groups: 4.75 ± 4.06 in the app-based education program group, 1.39 ± 3.58 in the electronic document group, and −1.11 ± 6.31 in the control group (F = 6.254, *p* = 0.004). The posttest found that the app education program scored higher for the differences in parenting confidence than the control group. Therefore, Hypothesis 3 “parenting confidence will differ among the app-based program, electronic document, and control groups” was supported.

**Figure 6:**
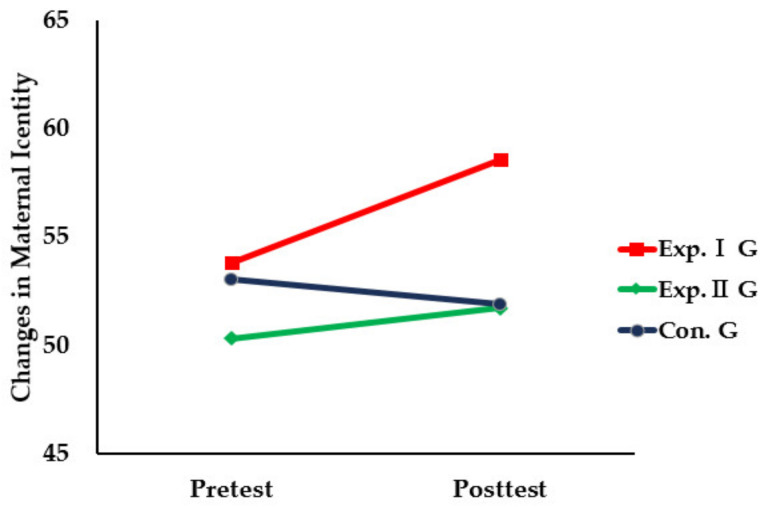
Comparison of changes in maternal confidence among three groups.

The results of the comparison of parenting confidence among the three groups before and after the intervention using the app-based parenting support education program for premature infants’ mothers were as follows (Table 6, Figure 6).

#### 3.2.4. Hypothesis 4

“Satisfaction with education will differ among the app education program and the electronic document groups”.

For satisfaction with education, the participants scored 32.54 ± 1.81 in the app-based education program group and 31.06 ± 1.86 in the electronic document group. There were significant inter-group differences in satisfaction with education (*t* = 2.222, *p* = 0.034). Therefore, Hypothesis 4 was supported.

The results of the comparison of satisfaction with education among the two groups before and after the intervention using the app-based parenting support education program for premature infants’ mothers were as follows (Table 7).

## 4. Discussion

### 4.1. Development of App-Based Parenting Support Program

This study utilized a nonequivalent control group non-synchronized design to develop an app-based program for mothers of premature infants. The program is based on Mercer’s (1981) maternal role attainment model [39], and the study aimed to determine its effects on these mothers.

Education using a smartphone app, which is characterized by accessibility and convenience, can be used without temporal or spatial restrictions and its users can be given an immediate chance to partake in education at any time. As the worldwide COVID-19 pandemic and many other types of infectious disease have activated noncontact education, development of an app-based education program can be applicable to premature infants’ parents as an effective way of providing them with information.

Because the parenting roles are important for premature infants at each stage of their growth and development from post-birth institutionalization in the NICU through to infanthood and early childhood, it is necessary to develop a sustainable and systematic program that meets each development stage [40]. So, the teaching contents were formed to cover the stay in hospital and also the early period of parenting at home. Specifically, the app contains general teaching contents regarding medical devices applied to premature infants during their stay in the NICU, their growth and development, kangaroo care, and the programs for supporting premature infants; an area to record feeding, defecation frequency, and growth status (height, weight, head circumference); a parenting diary-keeping zone for emotional support; and a professional counseling center enabling two-way communication with the researcher.

The existing research confirming the effectiveness of systematic education in premature infants’ mothers [37,41] has demonstrated that the better understanding the mothers had of premature infants’ physical and physiological characteristics and development, the less parenting stress they experienced and the higher levels of parenting confidence and maternal attachment they showed. In a different way from the discharge education program that informs on basic parenting skills, this study aimed to support an understanding of premature infants and help parents to participate in their care from the moment of staying in the NICU. Based on the findings of the literature review that parents of premature infants in NICUs might experience emotional distress, which involves symptoms of post-traumatic stress disorder and postpartum depression [7,42,43], and that emotional support reduced their stress and raised their parenting confidence and maternal attachment [37], the mothers were asked to keep a parenting diary so that they could feel comfortable and become confident in and satisfied with their performance of their maternal role.

An online parenting community is a principal variable that allows users with many different types of interests in pregnancy, childbirth, and education to engage in communication [44]. A parenting-centered communicative means can reduce parenting stress and assist with emotional support [45]. However, most of the existing studies employed no communication [23,25]. So, a professional counseling center that enables two-way communication with the researcher was established to allow for sharing information and opinions in addition to counseling for emotional support. A membership community was also created for inter-user two-way communication. This helped premature infants’ mothers share their daily life, be given systematic information about their babies, and participate actively in baby care from the early stage of parenting through to childrearing.

While the existing education programs for premature infants’ mothers were limited to one-time programs, which involved face-to-face interviews, telephone interview and handouts, this study enabled them to use information efficiently without temporal or partial restrictions and store data with ease, as smartphone education has recently seen an uptick. Hospitals and communities are expected to provide diverse education programs, and with a smartphone distribution rate of 96% in South Korea now, as well as various advantages of an app-based education program, its use seems beneficial in premature infant care.

### 4.2. Effects of App-Based Parenting Support Program

After the educational intervention, both the app-based education program and electronic document groups had higher levels of maternal identity, maternal postpartum attachment, and parenting confidence than the control group. This is consistent with the finding that a web-based education program, which was developed for and applied to premature infants’ mothers, was effective in increasing maternal identity, maternal attachment, and parenting confidence and in reducing postpartum depression and anxiety [41]. It is also consistent with the findings of the study that developed the NICU2HOME app for parents of premature infants leaving hospital, which improved parenting confidence and self-efficacy after the intervention [46]. These results may be since the app-based education program can be used freely any time without temporal or spatial restrictions, instead of the existing one-time education, from the post-birth stay in hospital through to the entire course of parenting at home. It can be used to record the daily routines of the baby (feeding, defecation, height, weight, head circumference) and keep a parenting diary, as well as receive technical knowledge and counseling regarding parenting, which supports mothers to assume their maternal role.

The finding that premature infants’ mothers using the app-based education program saw their parenting confidence significantly increase, when compared with the other two groups, indicates that applying the education program from the early stage of parenting can help improve their parenting confidence, even taking the short period of the two-week study intervention into account.

In telephone interviews after undertaking the education program, both the app-based education program and electronic document group members answered that using visual materials, including images and videos, improved their understanding of education and raised their interest in learning, more so than using texts. The app-based education program group positively evaluated the technicality of information and accessibility and convenience of the app-based program. In particular, they answered that in terms of information, the contents related to premature infant support programs and preparations in kangaroo care were useful. Previously, research on the experience among parents of low-birth-weight infants of using social networks [47], and a study that induced premature infants’ parents to participate in the development of a digital education program [48], obtained similar results.

The electronic document group saw a significant increase in maternal identity and maternal postpartum attachment, when compared with the control group. To put these results and the findings of the literature review together, in future work, it is necessary to identify education media desired by users and their education needs and then, based on the findings, apply an intervention program. It may also be effective in terms of education giving teaching materials in an electronic form if there are recipients who can hardly use a smartphone.

This study has confirmed that an app-based education program is more likely to ensure premature infants’ mothers efficiently receive full education than the existing types of face-to-face or written education. Because premature infants’ mothers require more knowledge and skills in parenting than those of term infants, it is vital to give long-term, regular education, instead of a one-time education program. In particular, mothers’ recovery from premature childbirth or their infants’ ill-health can affect their participation in education and make it difficult for them to attend regular face-to-face education; therefore, smartphone use of an app-based education program can be highly suitable for premature infants’ mothers.

A few limitations of the present study must be noted. First, this study design failed to randomize the sample to prevent the experiment being compromised by communication between groups, and the subjects themselves perceived their participation in the experiment. Due to the possibility of these issues affecting the results, in future work, it is necessary to consider how to control for these factors and thereby improve the quality of the study. Second, this study had restrictions in its sampling because premature infants with congenital malformations or severe complications and their mothers were excluded. Further research needs to develop interventions for mothers of high-risk neonates, as well. Third, the difficulty in sampling due to the subjects’ characteristics made it impossible to meet the estimated sample size. To overcome this problem, further research needs to extend the subjects included when testing the program for its effectiveness.

## 5. Conclusions

These results have demonstrated that the parenting support education program for premature infants’ mothers was effective in improving maternal identity, maternal postpartum attachment, and parenting confidence.

As South Korea has an all-time low birthrate but premature infants are increasing each year, applying a parenting support education program for premature infants’ mothers is important, both to support them to assume their maternal role and so that premature infants grow healthier. Premature infants’ childrearing should no longer be a concern restricted to their parents and medical staff, but instead an area of general social concern that requires for improvements to the existing education and support.

Smartphone use of an app-based education program is a useful educational means readily available to users, whereby they can learn and interact with experts any time, without temporal or spatial restrictions. Since it can also give mothers access to diverse technical information from the infants’ stay in hospital after birth through to care at home, the program is expected to be of great help to mothers in terms of infant care.

On the basis of these results, the following suggestions can be made: Because there are only a few app-based education programs for premature infants’ mothers, it is necessary to conduct studies of this kind repeatedly with the aim of such programs being validated. Further research also needs to be conducted to determine the efficiency of app-based education programs through a prolonged intervention with the objective of confirming the effects on premature infants’ growth and development. Linked to that point, it is necessary to develop a research tool for assessing premature infants’ growth and development after applying an app-based education program. Finally, taking the strengths of the app-based education program into account, it is also advisable to investigate how such a program can be applied in association with communities and other relevant institutions beyond primary medical institutions such as hospitals.

## Figures and Tables

**Table 1:** Homogeneity test of general characteristics of premature infants’ mothers.

Variables	Categories	Exp. Ⅰ (N = 16)	Exp. Ⅱ (N = 18)	Cont. (N = 18)	χ^2^ or F	*p*
		N (%) or M ± SD	N (%) or M ± SD	N (%) or M ± SD		
Age (years)	25~30	0 (0.0%)	1 (5.6%)	2 (11.1%)	9.437	0.051
	31~35	8 (50.0%)	12 (66.7%)	15 (83.3%)		
	36~40	8 (50.0%)	5 (27.8%)	1 (5.6%)		
Education	≤High School	0 (0.0%)	0 (0.0%)	1 (5.6%)	1.926	0.382
	College	16 (100.0%)	18 (100.0%)	17 (94.4%)		
Monthly income	250~399	1 (6.3%)	5 (27.8%)	2 (11.1%)	0.914	0.407
	400~549	7 (43.8%)	4 (22.2%)	4 (22.2%)		
	≥550	8 (50.0%)	9 (50.0%)	12 (66.7%)		
		587.50 ± 183.98	542.22 ± 207.02	644.44 ± 203.56	0.619	0.543
Number of children	1	10 (62.5%)	11 (61.1%)	15 (83.3%)	2.578	0.276
	≥2	6 (37.5%)	7 (38.9%)	3 (16.7%)		

Exp. = Experimental Group; Cont. = Control Group.

**Table 2:** Homogeneity test of general characteristics of premature infants.

Variables	Categories	Exp. Ⅰ (N = 16)	Exp. Ⅱ (N = 18)	Cont. (N = 18)	χ^2^ or F	*p*
		N (%) or M ± SD	N (%) or M ± SD	N (%) or M ± SD		
Gestational age (day)		244.06 ± 8.87	240.44 ± 13.78	233.78 ± 13.72	3.012	0.058
Period of hospitalization (day)		15.94 ± 8.87	12.89 ± 6.02	15.94 ± 6.80	1.041	0.361
Gender	Boy	7 (43.8%)	6 (33.3%)	5 (27.8%)	0.975	0.614
	Girl	9 (56.3%)	12 (66.7%)	13 (72.2%)		
Delivery type	Vaginal	4 (25.0%)	9 (50.0%)	9 (50.0%)	2.836	0.242
	Cesarean	12 (75.0%)	9 (50.0%)	9 (50.0%)		
Oxygen therapy	Yes	12 (75.0%)	9 (50.0%)	8 (44.4%)	3.578	0.167
	No	4 (25.0%)	9 (50.0%)	10 (55.6%)		
Ventilator therapy	Yes	7 (43.8%)	8 (44.4%)	7 (38.9%)	0.134	0.935
	No	9 (56.3%)	10 (55.6%)	11 (61.1%)		
Weight (gm)		2266.88 ± 339.90	2041.67 ± 362.35	2143.27 ± 249.90	0.032	0.969
Height (cm)		44.06 ± 1.97	45.16 ± 2.66	44.94 ± 2.96	2.103	0.133
Head circumference (cm)		31.51 ± 0.97	30.34 ± 1.93	30.64 ± 1.04	3.155	0.051

Exp. = Experimental Group; Cont. = Control Group.

**Table 3:** Homogeneity test of dependent variable among three groups.

Category	Exp. Ⅰ (N = 16)	Exp. Ⅱ (N = 18)	Cont. (N = 18)	F	*p*
	M ± SD	M ± SD	M ± SD		
Maternal identity	58.88 ± 5.18	51.39 ± 13.40	61.67 ± 7.14	5.735	0.006
Maternal postpartum attachment	59.84 ± 4.09	56.39 ± 9.36	61.48 ± 3.41	3.056	0.056
Maternal confidence	53.81 ± 8.78	50.33 ± 8.15	53.06 ± 6.49	0.949	0.394

Exp. = Experimental Group; Cont. = Control Group.

**Table 4:** Comparison of changes in maternal identity among three groups.

Variables	Categories	Exp. Ⅰ (N = 16)	Exp. Ⅱ (N = 18)	Cont. (N = 18)	F	*p*	F(*p*) *
		M ± SD	M ± SD	M ± SD			
Maternal identity	Pre	58.88 ± 5.18	51.39 ± 13.40	61.67 ± 7.14	5.735	0.006	1.550(0.223)
	Post	62.56 ± 6.00	55.61 ± 14.57	61.00 ± 6.33	2.346	0.106	
	Difference	3.69 ± 4.09	4.22 ± 8.13	−0.67 ± 6.84	2.884	0.065	

Exp. = Experimental Group; Cont. = Control Group. * ANCOVA (covariate values: pre-maternal identity = 57.25.

**Table 5:** Comparison of changes in maternal postpartum attachment scale among three groups.

Variable	Categories	Exp. Ⅰ (N = 16) ^a^	Exp. Ⅱ (N = 18) ^b^	Cont. (N = 18) ^c^	F	*p* Scheffe
		M ± SD	M ± SD	M ± SD		
Maternal postpartum attachment scale	Pre	59.84 ± 4.09	56.39 ± 9.36	61.48 ± 3.41	3.056	0.056
	Post	63.93 ± 3.57	60.28 ± 6.89	61.69 ± 4.58	2.064	0.138
	Difference	4.09 ± 3.72	3.88 ± 5.02	0.22 ± 3.69	4.716	0.013^a^, ^b^ > ^c^

Exp. = Experimental Group; Cont. = Control Group. ^a^ = Experimental Group I; ^b^ = Experimental Group II; ^c^ = Control Group.

**Table 6:** Comparison of changes in maternal confidence among three groups.

Variable	Categories	Exp. Ⅰ (N = 16) ^a^	Exp. Ⅱ (N = 18) ^b^	Cont. (N = 18) ^c^	F	*p* Scheffe
		M ± SD	M ± SD	M ± SD		
Maternal confidence	Pre	53.81 ± 8.78	50.33 ± 8.15	53.06 ± 6.49	0.949	0.394
	Post	58.56 ± 5.72	51.72 ± 6.53	51.94 ± 5.19	7.354	0.002^a^ > ^b^, ^c^
	Difference	4.75 ± 4.06	1.39 ± 3.58	−1.11 ± 6.31	6.254	0.004^a^ > ^c^

Exp. = Experimental Group; Cont. = Control Group. ^a^ = Experimental Group I; ^b^ = Experimental Group II; ^c^ = Control Group.

**Table 7:** Comparison of satisfaction between app program and learning with PDF group.

Variable	App program (N = 16)	PDF learning (N = 18)	*t*	*p*
	M ± SD	M ± SD		
Satisfaction	32.54 ± 1.81	31.06 ± 1.86	2.222	0.034

## Data Availability

The data that support the findings of this study are available from the corresponding author upon reasonable request.
